# Comparison between two anammox fiber fillers under load impact and the effect of HCO_3_^−^ concentration

**DOI:** 10.1039/d1ra07982d

**Published:** 2021-12-20

**Authors:** Weiqiang Wang, Jinghai Zhu, Deqi Xiong, Yang Su, Yehui Li, Jinxiang Fu

**Affiliations:** College of Environmental Science and Engineering, Dalian Maritime University 116026 Dalian China; School of Municipal and Environmental Engineering, Shenyang Jianzhu University 110168 Shenyang China fujinxiang@sina.com

## Abstract

Based on the establishment of a stable anaerobic ammonia oxidation treatment system in 100 days, the impact resistances of two different anammox fiber fillers (the curtain filler: R1 and the bundle filler: BR) were compared. Furthermore, the effect of HCO_3_^−^ concentration on the bundle filler system was also investigated, the results have shown that the activity of the two anammox fiber fillers was not inhibited when the NO_2_^−^–N concentration was lower than 750 mg L^−1^ (FNA = 0.085 mg L^−1^), while it was significantly suppressed at 900 mg L^−1^ (FNA = 0.118 mg L^−1^). However, the two fiber fillers could be recovered and exhibit a good impact resistance reduction of the substrate concentration. On day 95, the structure of the bundle filler was more conducive to the stable attachment, proliferation, and aggregation of anammox bacteria. Dominant anammox bacteria in both the curtain and bundle fillers were *Candidatus* Kuenenia, which accounted for 25.9% and 35.9% of the total population, respectively. When the influent HCO_3_^−^ concentration was 900 mg L^−1^, the bundled fiber filler had the highest total nitrogen (TN) removal efficiency, which reached 89.0%. Even though it was inhibited under 2000 mg L^−1^ of HCO_3_^−^ concentration, the reactor was able to recover within one week by reducing the substrate concentration. In addition, the HCO_3_^−^ inhibition mechanism was independent of pH, which resulted in high FA content.

## Abstract 2

Based on the establishment of a stable anaerobic ammonia oxidation treatment system in 100 days, the impact resistances of two different anammox fiber fillers (the curtain filler: R1 and the bundle filler: BR) were compared.

## Graphical abstract

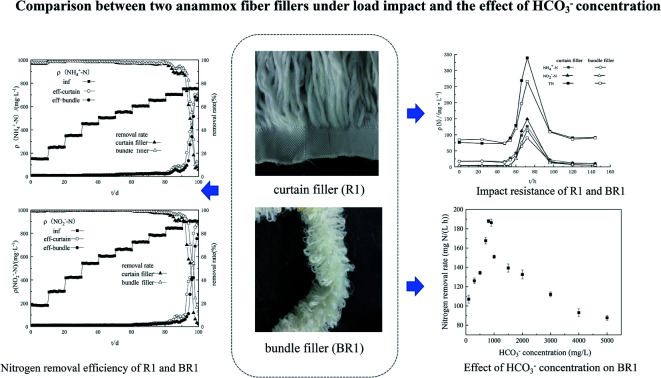

## 1. Introduction

The anaerobic ammonia oxidation (anammox) process has a high nitrogen removal rate, a low sludge yield, and no need for additional organic carbon requirements.^1^ However, the doubling time of anammox bacteria is about two weeks, and the lack of biomass limits further application worldwide.^2^ To maintain a high anammox biomass concentration in the reactor, the formation of granular sludge or biofilm was found to be a feasible and effective method. The granular sludge reactor has higher volatile suspended matter and its nitrogen removal efficiency is higher than that of the biofilm reactor. However, the cultivation of granular sludge requires a long time. Even during stable operation, the granular sludge may be unsteady, and the reactor can show a floating sludge problem.^3–5^ So far, there is still a controversy about the composition of biofilms in general. Among these, the most consistent view is that biofilms are composed of loosely distributed amorphous microbial communities under microbial aggregates, including adsorbed substrates and inorganic particles with extracellular polymers surrounding them.^6–8^ The biofilm provides a large specific surface area, strong adsorption capacity, and adequate environment suitable for the enrichment of anammox bacteria.^9^ Due to the compact nature of the microbial biofilm surface, the anammox process has significant advantages.^10^ Many studies have investigated the substrate removal capacity and retention ability of different anammox biofilms made of volcanic rock, zeolite, ceramics, and plastic.^11^ However, few studies have focused on the comparison between different types of anammox biofilms.

Autotrophic bacteria use inorganic carbon as their carbon source (HCO_3_^−^).^12^ Sufficient inorganic carbon is needed to enrich anammox bacteria and retain their activity.^13^ It has been reported that the activity of anammox bacteria increased as the influent inorganic carbon concentration increased from 1.0 g L^−1^ to 1.5 g L^−1^, but was inhibited at 2.0 g L^−1^.^14^ Additionally, excessive concentrations of ammonia form FA (free ammonia) in water, and the pH leads to an increase in FA in the environment.^15^ Therefore, the inhibition of the activity of anammox bacteria caused by high HCO_3_^−^ concentrations and FA is possible in nitrogen-containing wastewater. However, in the biofilm system, whether the HCO_3_^−^ concentration aggravates the inhibition of FA under high influent NH_4_^+^–N needs to be studied urgently.

Therefore, in this study, two different fiber fillers were selected to study the effect of substrate concentrations on the anammox biofilm. First, the impact resistance and recovery ability of the two fiber fillers were compared. The characteristics and community structure of the biofilms were also analyzed to provide information for practical applications. Moreover, the effect of HCO_3_^−^ concentration on the nitrogen removal rate and whether the inhibition of HCO_3_^−^ concentration is related to pH and FA were explored.

## 2. Materials and methods

### 2.1. Reactor configuration and operational strategy

#### 2.1.1 Impact load resistance and recovery test

**Fig. 1:**
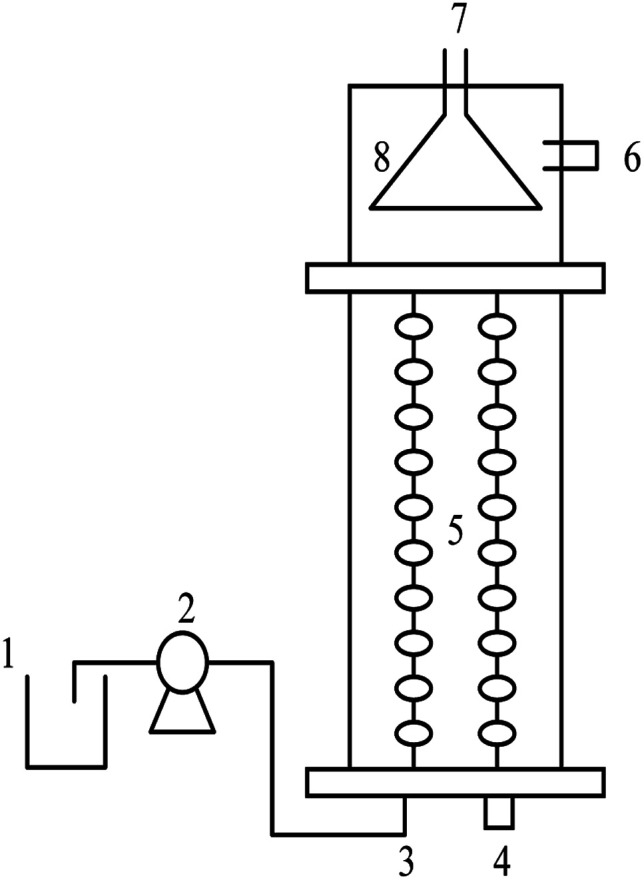
Up-flow biofilm reactor and process flow chart. 1. Water distribution tank; 2. metering pump; 3. intake; 4. auxiliary intake; 5. filler; 6. water outlet; 7. air outlet; 8. three-phase separator.

The experiment devices are shown in Fig. 1. Two same up-flow biofilm reactors with an effective volume of 20 L were operated for 100 days. There were two partitions inside the reactor: the reaction zone and separation zone. The inoculated sludge was taken from the reserve flocculent anammox sludge in the laboratory, and the total nitrogen removal efficiency was beyond 80%. The initial inoculated sludge concentration was about 3800 mg L^−1^ in two reactors. Additionally, the initial NH_4_^+^–N and NO_2_^−^–N concentrations were set as 150 mg L^−1^ and 180 mg L^−1^ respectively. The substrate concentration was increased for 9 times in 100 days. For the first four times, NH_4_^+^–N and NO_2_^−^–N concentrations were increased by 100 mg L^−1^ and 120 mg L^−1^, and for the last five times, they were increased by 50 mg L^−1^ and 60 mg L^−1^. Each stage lasted for 10 d. The pH value of influent water was controlled at 7.2 ± 0.2. The HRT was set as 6 h. The temperature was controlled at 32 ± 2 °C by the heat pipe heating equipment. To maintain anaerobic conditions and prevent the growth of phototrophic organisms, the reactor was wrapped with a black opaque cloth.^16^ Although a concentration of NH_4_^+^–N lower than 1000 mg L^−1^ cannot inhibit the anammox process,^17^ anammox bacteria were sensitive to the NO_2_^−^–N concentration.^18^ Therefore, the NO_2_^−^–N concentration in the effluent was used to evaluate the activity of anammox bacteria by controlling the temperature and pH.

#### 2.1.2 Influence of the concentration of HCO_3_^−^

The bundle filler reactor was selected to study the effect of HCO_3_– concentration on the TN removal rate, and NaHCO_3_ was added as the only source of HCO_3_^−^ for 70 d. Other operating parameters were the same as mentioned in Section 2.1. At the same time, the total nitrogen concentration in the influent was set to 1320 mg L^−1^, and the NaHCO_3_ concentration in the influent was increased in a gradient, as shown in Table 1.

**Table 1:** NaHCO_3_ concentration in different stages

Stage	Time	Concentration (mg L^−1^)
I	1–46	300–1100
		100/5 d
II	47–52	1500
	53–58	2000
	59–63	3000
	64–70	900

### 2.2. Fiber filler

**Fig. 2:**
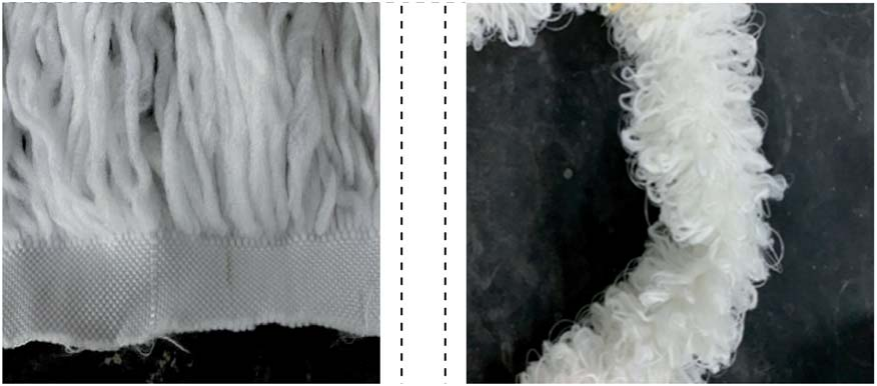
The fiber fillers in the reactors. (a) curtain filler, (b) bundle filler.

The fixed curtain filler and fixed beam filler in the experiment are shown in Fig. 2. Both fillers were made of fiber. The curtain filler (Jingyuan Environmental Technology Co., Ltd China) was made of acrylic fiber and polyester fiber. It could use a large amount of biological mass attached to its surface for oxygenation and repeated contact with sewage. Therefore, it could intercept tiny, suspended solids and organic matter that were not easy to precipitate or remove. The bundled filler (Jingyuan Environmental Technology Co., Ltd China) was composed of acrylic fiber, polyester fiber and reinforcing wire. The filler had a good biofilm formation, compact structure with no deformation, and strong resistance to water flow impact. Both fillers were suitable for the treatment of high nitrogen-containing wastewater.

### 2.3. Synthetic wastewater

NH_4_Cl and NaNO_2_ were used as the nitrogen source of the influent, for which NH_4_^+^–N : NO_2_^−^–N was 1 : 1.2. The other compositions of synthetic wastewater are as follows: NaHCO_3_ 1 g L^−1^; KH_2_PO_4_ 0.01 g L^−1^; MgSO_4_ 0.3 g L^−1^; and CaCl_2_ 0.056 g L^−1^. 1 mL L^−1^ of trace element concentrate I and II were added, according to Table 2. Nitrogen gas (99%) was purged into the reactor for 20 min to reduce the dissolved oxygen (DO) concentration till it was lower than 0.5 mg L^−1^.

**Table 2:** The composition of trace elements^19^

Solution	Composition	Concentration (mg L^−1^)
Trace element I	EDTA	5000
	FeSO_4_	5000
Trace elements II	EDTA	15 000
	ZnSO_4_·7H_2_O	430
	MnCl_2_·4H_2_O	990
	H_3_BO_4_	14
	CuSO_4_·5H_2_O	250
	NaMoO_4_·2H_2_O	220
	NaSeO_4_·10H_2_O	210
	NiCl_2_·6H_2_O	190

### 2.4. Inhibition pathway of HCO_3_^−^

The batch test was carried out with a series of serum bottles to investigate HCO_3_ inhibition, which is independent of pH. The same operational conditions as the bundle filler reactor were selected and different HCO_3_^−^ concentrations (100, 300, 500, 700, 900, 1500, 2000, 3000, 4000, and 5000 mg L^−1^) were used for batch experiments. The effective/total volume of each serum bottle was 150/165 mL. The serum bottle had an inner cap with a butyl rubber stopper and a plastic outer cap with a hole on the top. Each serum bottle was added with 10 g biofilm and 120 mL synthetic wastewater. The pH value was adjusted to 7.2 ± 0.2. The serum bottles were sparged with high-purity nitrogen for 20 min to form a dissolved oxygen-free environment. Then, the bottles were sealed and placed in an incubator at a constant temperature of 32 °C under dark conditions.^20^ The speed was set as 140 rpm. On day 6, day 8, and day 10 after stable operation, each sample was collected 6 times, each sample was collected 6 times, once an hour, and the average removal rate of TN was calculated to determine the maximum TN removal rate.

### 2.5. Chemical and physical analysis

NH_4_^+^–N and NO_2_^−^–N were estimated according to the standard methods.^21^ The methods of measurement and instruments are given in Table 3. The above-mentioned samples were measured in duplicate to guarantee the accuracy. The morphology of fiber fillers was observed using an E400 light microscope (Nikon Corporation, Japan).

**Table 3:** The methods of measurement and instruments

Item	Measure methods	Instrument
NH_4_^+^–N	Nessler's reagent method	
NO_2_^−^–N	*N*-1-Naphthyl-ethylenediamine method	Spectrophotometer
TN	Digestion of potassium persulfate	(WFJ 2100)
DO	LDO electrode fluorescence	Dissolved oxygen meter (HQ, 30d)
pH	Glass electrode method	ZD-2 automatic potentiometer
T		Thermometer

### 2.6. DNA extraction and illumina high-throughput sequencing

To compare the differences in microbial diversity between the two fiber filler systems, an appropriate amount of biofilm was taken from the reactors (the sampling port was located at 20 cm from the bottom of the reactor) on day 95. The collected samples were subjected to high-throughput analysis (curtain filler: R1; bundle filler: BR1). Total community genomic DNA extraction was performed using an E. Z. N. A.Soil DNA Kit (Omega, USA), following the manufacturer's instructions. To ensure that adequate amounts of high-quality genomic DNA were extracted, the concentration of DNA was measured using Qubit 2.0 (life, USA). The V3–V4 hypervariable region of the 16S rRNA gene was amplified using standard protocols. Sequencing was performed using the Illumina MiSeq system (Illumina MiSeq, USA) by Sangon BioTech Company (Shanghai China).

### 2.7. Calculation formula of FA and FNA

The calculation formula of FA and FNA (free nitrous acid) concentrations are as follows:^22^1
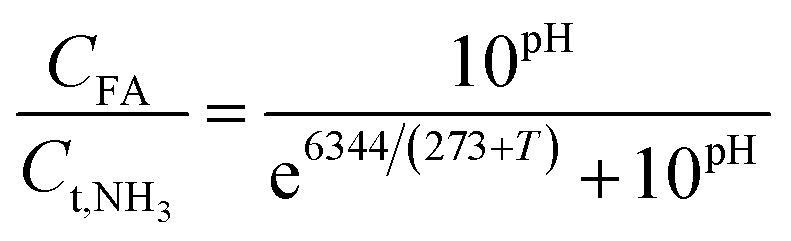
2
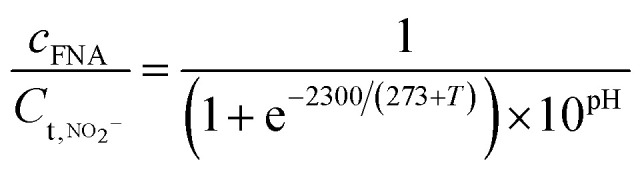
*C*_FA_ is the concentration of free ammonia, mg L^−1^; *C*_FNA_ is the concentration of FNA, mg L^−1^; *C*_t,NH_3__ is the concentration of total ammonia, mg L^−1^; *C*_t,NO_2_^−^_ is the concentration of total nitrite, mg L^−1^; *T* is the temperature in the system, °C. The pH value was controlled at 8 ± 0.2, and the temperature was controlled at 32 ± 2 °C.

## 3. Results and discussion

### 3.1. Nitrogen removal efficiency under different substrate concentrations

**Fig. 3:**
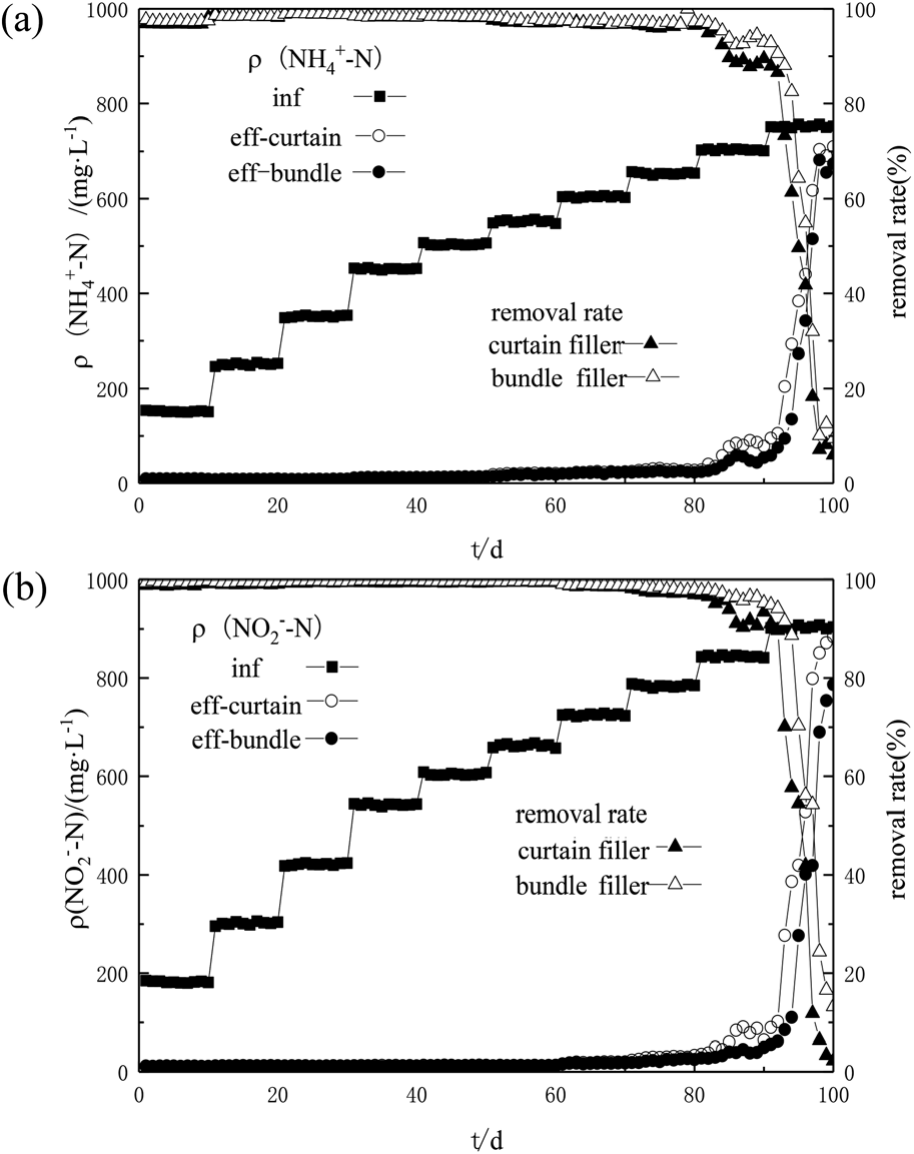
Effect of increasing NH_4_^+^–N (a) and NO_2_^−^–N (b) concentration on the nitrogen removal efficiency of the two fiber fillers.

With the increased NH_4_^+^–N and NO_2_^−^–N concentrations, the nitrogen removal efficiency of the two fillers increased at first and then decreased (Fig. 3). Until the substrate concentration increased to 990 mg L^−1^, NO_2_^−^–N in the system was almost exhausted and the removal rate of NO_2_^−^–N was stabilized at 99%. When the influent TN concentration reached 1320 mg L^−1^ and the FNA reached 0.085 mg L^−1^, the nitrogen removal performance of the two fillers slightly decreased to about 96% but not inhibited. In the early stage, it could be found that the recovery rate of the bundle packing was better than that of the curtain packing. While in the other sludge system, at this substrate concentration, nitrogen removal rates decreased 90% within 7 d.^23^ Moreover, different from slowly deteriorated nitrogen removal efficiency, many studies found that the substrate had caused severe inhibition of anammox activity, and the nitrogen removal efficiency deteriorated significantly.^24,25^ Since high substrate concentration would inevitably bring a high level of FA and FNA, which would inhibit the nitrogen removal efficiency. However, there was no obvious inhibition observed in the reactor, and it suggested that the anammox fiber fills have a high tolerance at a concentration of 1320 mg L^−1^. When the total nitrogen concentration gradually increased to 1540 mg L^−1^, the nitrogen removal efficiency of the bundle filler was better than that of the curtain filler, and the removal rates of NH_4_^+^–N and NO_2_^−^–N were 87% and 89%, which indicated that the bundle filler had better resistance to high NO_2_^−^–N concentrations. On day 90, when the concentration increased to 1650 mg L^−1^, the two reactors were inhibited and gradually deteriorated. The removal rates of NH_4_^+^–N and NO_2_^−^–N were both less than 20% and the effluent NO_2_^−^–N concentration was more than 700 mg L^−1^.

Nitrite is a vital factor affecting the activity of anammox bacteria.^26^ Excessive nitrite would form FNA in the liquid, which directly affected the crucial enzymes of microbial metabolism and stimulated the accumulation of intermediate products, thereby inhibiting the metabolism of anammox bacteria.^27^ In this study, when the NO_2_^−^–N concentration was lower than 750 mg L^−1^ (FNA = 0.085 mg L^−1^), the activity of the both the anammox fiber fillers was not inhibited. Comparing these results with other studies,^28–30^ the threshold value of NO_2_^−^–N in this reactor was higher. Meanwhile, the FNA threshold value of granular sludge and gel carrier was higher than that of flocs. It was speculated that the biofilm thickness or particle size plays an important role in NO_2_^−^–N threshold value. When the influent NO_2_^−^–N concentration reached 900 mg L^−1^ (FNA = 0.118 mg L^−1^) for 12 h, the activity of anammox bacteria in the reactor decreased significantly, and foam appeared in the top area of the reactor. These phenomena could indicate that some anammox bacteria died under high NO_2_^−^–N concentrations.^21^ This study showed that the anammox fiber fills provided suitable culture conditions for the growth and reaction of anammox bacteria and expanded the resistance range. Unfortunately, the high concentration of nitrite toxicity was still inevitable.

### 3.2. Comparison of the impact resistance between two fiber fillers

**Fig. 4:**
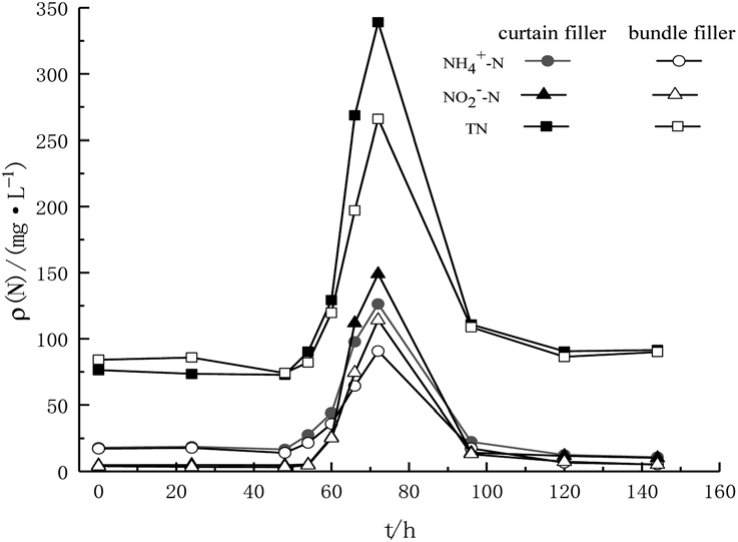
Nitrogen content in the effluent and impact resistance of the two kinds of fillers.

The two fiber filler systems stably operated when the influent TN concentration was 770 mg L^−1^ (Fig. 4). It proved that even though the nitrogen removal efficiency decreased, the fiber filler system still had high activity after recovery by a decreased substrate concentration. After the influent TN concentration increased to 1430 mg L^−1^ in the first 12 h, there was no significant difference in the removal efficiency between the two kinds of fillers. The NH_4_^+^–N concentration in the effluent was 44.0 mg L^−1^ and 35.9 mg L^−1^, and the NO_2_^−^–N concentration was 25.8 mg L^−1^ and 25.2 mg L^−1^, respectively. However, in the last 12 h of the cycle, the nitrogen removal efficiency of the two fiber fillers showed an obvious difference. The nitrogen efficiency of the bundle filler was more stable than that of the curtain filler, and the difference in total nitrogen concentration of the effluent was more than 70 mg L^−1^. In the subsequent recovery stage, the two fiber filler systems recovered rapidly within 24 h after the influent matrix concentration restored to 770 mg L^−1^. The NH_4_^+^–N/NO_2_^−^–N concentrations of the effluent of the curtain filler and bundle filler were 22.39/14.19 mg L^−1^ and 17.44/13.07 mg L^−1^, respectively. Within 1 d after recovery under low influent substrate concentrations, the NH_4_^+^–N concentration in the effluent of the bundle filler almost recovered to the value before the impact test, and the effluent NO_2_^−^–N concentration also decreased significantly. After only 3 d, the effluent NH_4_^+^–N and NO_2_^−^–N concentrations were reduced to below 15 mg L^−1^ and 11 mg L^−1^, respectively. The recovery of the bundle filler was faster than that of the curtain filler during the increase in substrate concentration.

### 3.3. Comparison of fiber fillers before and after biofilm formation

**Fig. 5:**
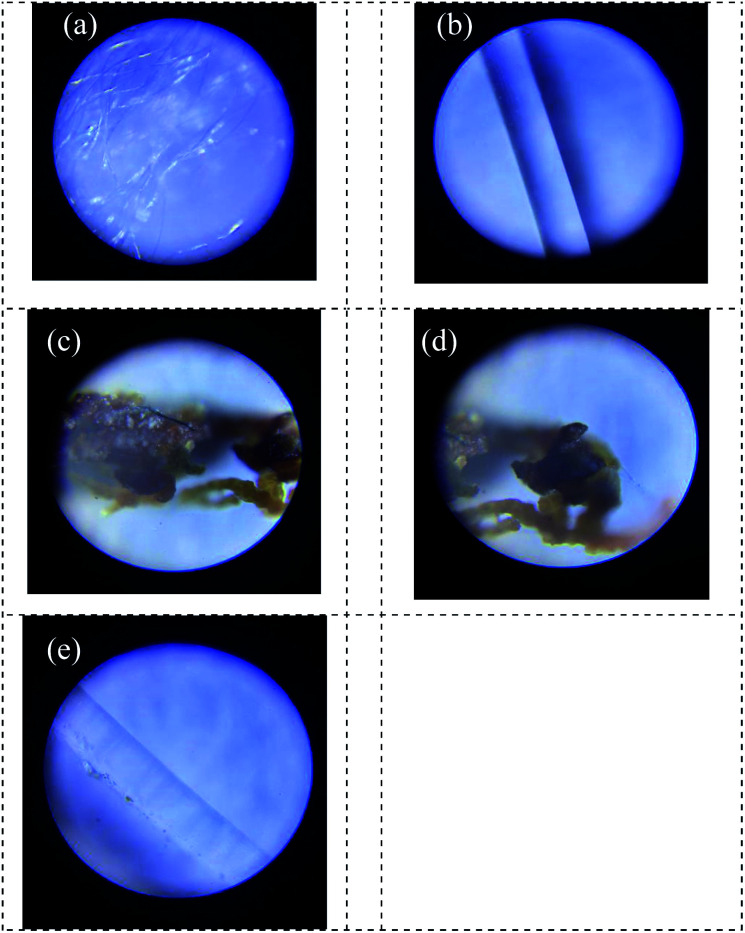
Microstructure before and after biofilm formation. (a) Before filler formation; (b) before reinforcing fiber formation; (c) after bundle filler formation; (d) after curtain filler formation; and (e) after the reinforcing fiber formation.

The two biofilm fillers were observed before and after the biofilm formation using an E400 light microscope (Nikon Corporation, Japan) on day 95. It could be seen from Fig. 5 that the surface of the fillers was clean and smooth before the anammox biofilm was hanging filmed. The fiber material (Fig. 5(a)) had a small and dense packing structure, which was helpful for trapping anammox bacteria, while the reinforced fiber material (Fig. 5(b)) had a smooth surface under the microscope, which was more conducive to supporting the fibrous packing. Fig. 5(c) and (d) show the microstructures of fiber biofilms. The entire biofilm was reddish-brown, which was consistent with the characteristics of anammox bacteria. Fig. 5(e) shows a kind of reinforcing fiber, with a small number of bacteria distributed on the surface, which proved that it also had some retention ability but was weaker than that of the fiber filler.^31^ Due to the adhesion and accumulation of bacteria on the fiber surface, the weight of the fiber increased. However, the reinforcing fiber was easy to be washed with water, resulting in a loose fiber structure. The curtain filler had a strong hydrophilic ability and large specific surface but was vulnerable to the impact of the influent and floating with the water flow fluctuation. After most bacteria adhered to the fiber surface, the weight of the fiber increased, and the fiber was easy to fracture with the water flow, and new fillers needed to replace regularly. The bundled filler was made of dense fibers with high toughness and had a large specific surface area. Therefore, many bacteria were attached to the bundled biofilm, and the fiber was not easy to fall off. Meanwhile, it had a certain tolerance to the erosion of water. In conclusion, the curtain filler and bundle filler were different in structure and application performance. Due to its meticulous structure, the bundle filler increased the specific surface area, which was conducive to improving the attachment of anammox bacteria. Moreover, filaments strengthened the toughness of the bundle filler, which could resist the scouring force of water flow and prevent the bacteria and fibers from falling off easily.

### 3.4. Microbial community structure and abundance of two fillers

**Fig. 6:**
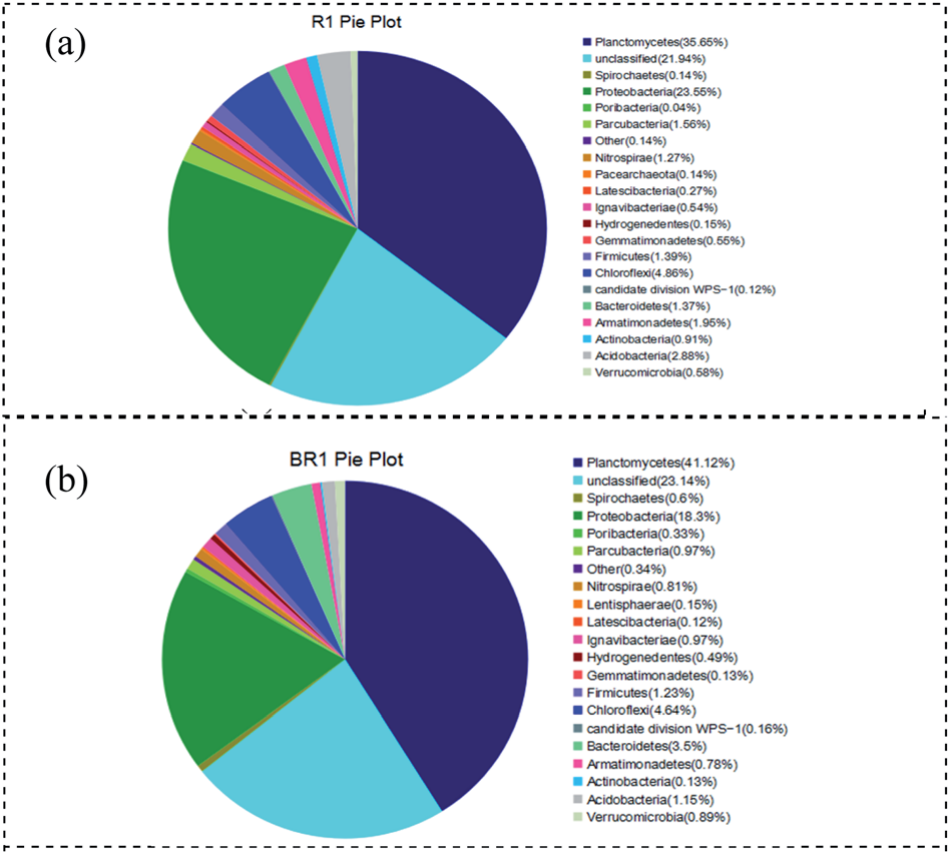
Frequency of microbial species at the level of phylum: (a) bundle filler and (b) curtain filler.

The phyla of curtain filler and bundle filler were compared (Fig. 6). *Proteobacteria* accounted for 18.3% of the total bacterial community in the bundled filler, which was 5.3% lower than that of the curtain filler. *Planctomycetes* (the phylum of anammox bacteria) in the bundle packing and curtain packing accounted for 41.1% and 35.7% respectively, which were higher than 15.8% in other studies.^32^ These results may explain that the anammox biofilm system was better than the activated sludge system with a high nitrogen concentration. Compared with the curtain filler, the bundle filler was more conducive to the growth and attachment of *Planctomycetes*. In addition, the difference in *Bacteroides* between the two filler samples was 2.1%. *Bacteroidetes* were chemoheterotrophic bacteria. Although no exogenous organic substances were added into the anammox reactor, the metabolic growth and reproduction of organisms in the systems would inevitably produce organic substances, increasing *Bacteroidetes*. Moreover, the bacteria belonging to *Chloroflexi* and *Bacteroidetes* have some impact on sludge granulation.

**Fig. 7:**
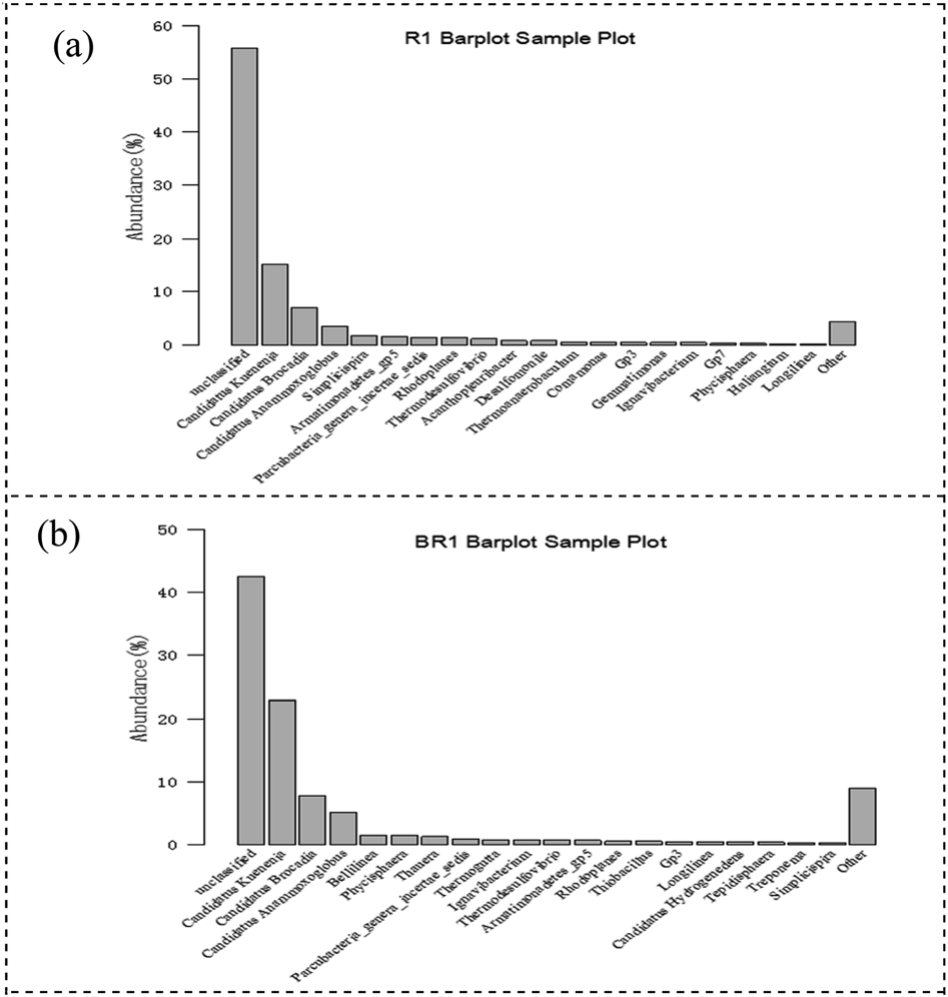
Frequency of microbial species at the level of genus: (a) bundle filler and (b) curtain filler.

Combined with Section 3.3, it could be seen from Fig. 7 that *Candidatus* Kuenenia, *Candidatus* Brocadia, and *Candidatus* Anammoxoglobus accounted for a high proportion in both curtain fillers and bundle fillers, and *Candidatus* Kuenenia dominated in the system, which gives an advantage for the anammox process. *Candidatus* Kuenenia could utilize low NO_2_^−^–N concentration and has good tolerance to high NO_2_^−^–N concentrations, which is consistent with the results of this study.^33^ The results of high-throughput sequencing also confirmed that the content of *Candidatus* Kuenenia in the bundle filler was higher than that in the curtain filler. The number and distribution of anammox bacteria on the surface of the curtain and bundle fillers were increased and evenly distributed, and the biofilm tended to mature. It was proved that the appropriate substrate concentration in the environment was conducive to the proliferation and aggregation of anammox bacteria. Moreover, it confirmed that the two kinds of fillers were suitable for nitrogen removal in wastewater, but bundle filler was better.

### 3.5. Effect of HCO_3_^−^ concentration on nitrogen removal performance

**Fig. 8:**
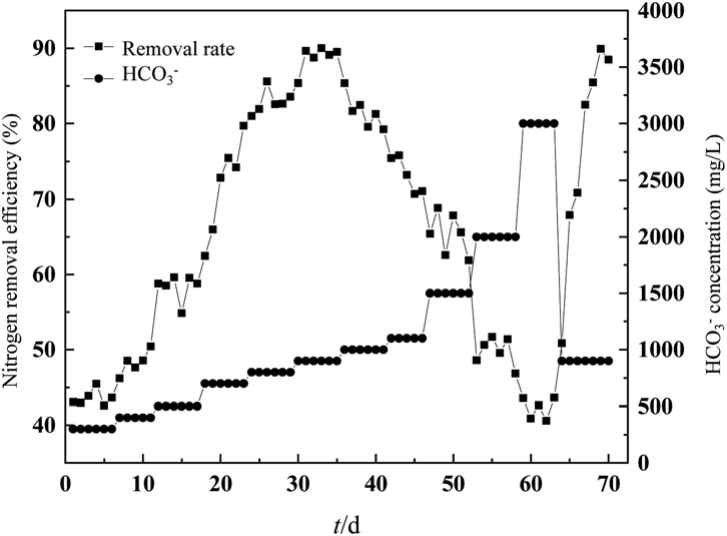
Variation in TN concentration in and out of the reactor at different HCO_3_^−^ concentrations.

To explore the optimum and inhibition value of HCO_3_^−^ concentration in the fiber filler system, the HCO_3_^−^ concentration gradually increased when the influent TN stabilized at 1320 mg L^−1^. It could be seen from Fig. 8 that when HCO_3_^−^ concentration was 300–700 mg L^−1^, the TN removal rate was below 80%. Until HCO_3_^−^ concentration reached 900 mg L^−1^, it had the highest TN removal rate (89.0%). In addition, at higher HCO_3_^−^ concentrations (1500 mg L^−1^), the concentration of FA reached 15 mg L^−1^ and the removal rate of TN decreased. Due to the adaption period of the increasing NH_4_^+^–N concentration, inhibition caused by FA was not easy to occur.

Studies have shown that when the HCO_3_^−^ concentration increased from 150 mg L^−1^ to 1500 mg L^−1^, the TN removal efficiency increased to twice the initial value,^34^ and the highest TN removal rate was 66.4 g N (m^3^ d)^−1^.^35^ After the HCO_3_^−^ concentration was further increased to 2000 mg L^−1^, the TN removal rate dropped to 50% within 5 d, and the effluent TN concentration increased to 650 mg L^−1^, which was lower than the inhibition level (75%) in other studies.^36^ However, once the HCO_3_^−^ concentration was reduced to 900 mg L^−1^ on day 64, the TN removal rate of the bundled fiber filler recovered to 85% in a short time, and the effluent NO_2_^−^–N concentration was lower than 20 mg L^−1^. It also reported similar bicarbonate inhibition of short recovery period.^37^ A hypothetical explanation proposed for this phenomenon was that the inhibited effect was due to high FA concentrations. However, HCO_3_^−^ concentration less than 1000 mg L^−1^ may cause the lack of inorganic carbon, further inhibiting the anammox process. For the fiber filler system, due to the lower HCO_3_^−^ consumption and the uniform distribution of bacteria, the HCO_3_^−^ inhibition concentration was lower. On the contrary, since anammox bacteria were in the core of the flocs, they could act better against the increasing HCO_3_^−^ concentration. Low HCO_3_^−^ concentrations could lead to a decrease in pH, resulting in the overflow of carbon dioxide during the aeration phase of the SBR. However, at a HCO_3_^−^ concentration of 1000 mg L^−1^, no bicarbonate limitation was observed in the reactor.

**Fig. 9:**
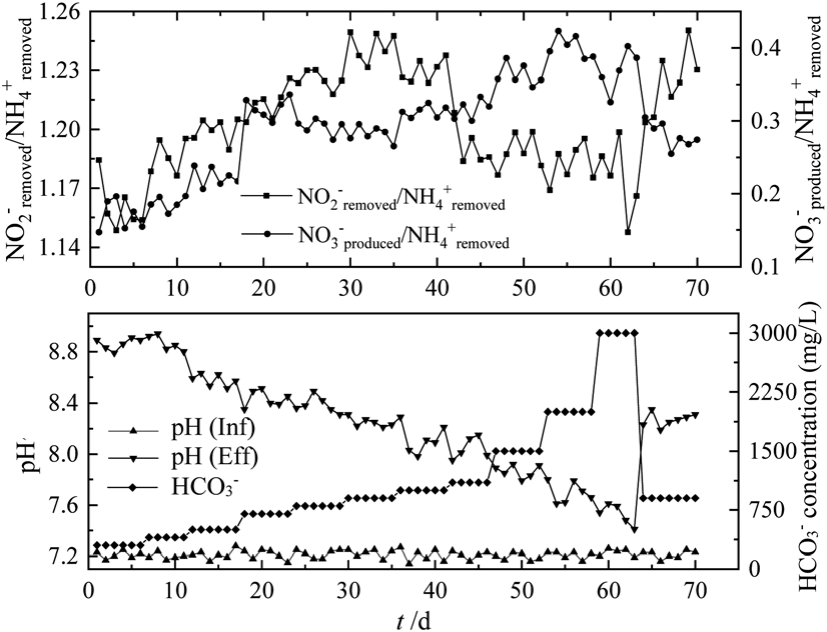
Effect of HCO_3_^−^ concentration on the stoichiometric ratio and pH in each stage.

The stoichiometric ratio could reflect the stability of the anammox process. As shown in Fig. 9, when the influent HCO_3_^−^ concentration was 900 mg L^−1^ (the best HCO_3_^−^ concentration), it had the highest nitrogen removal efficiency, and its NH_4_^+^_removed_/NO_2_^−^_removed_/NO_3_^−^_produced_ was 1/1.24/0.27, close to the theoretical value (1/1.32/0.26) in the anammox reaction. Yang reported that the stoichiometric ratio of NH_4_^+^_removed_/NO_2_^−^_removed_/NO_3_^−^_produced_ was 1 : 1.24 : 0.18 as inorganic carbon beyond 60 mg L^−1^.^37^

It was found that the optimal HCO_3_^−^ concentration was 1500 mg L^−1^, and the ratio of NH_4_^+^_removed_ to NO_2_^−^_removed_ was 1 : 1.28.^38^ Excessive or insufficient HCO_3_^−^ concentration would lead to excessive deviation of the reaction metering ratio. For influent HCO_3_^−^ concentrations of 300 mg L^−1^ and 700 mg L^−1^, NO_3_^−^_produced_/NH_4_^+^_removed_ was 0.18 and 0.30, respectively. Starting from exceeding the optimal HCO_3_^−^ concentration (900 mg L^−1^), the stoichiometric ratio ranged between 0.31 and 0.42, exceeding the theoretical value of 0.26. According to the anammox reaction process, every 1 mol of NH_4_^+^–N will consume 0.13 mol of H^+^ and 0.066 mol of HCO_3_^−^, which increased the pH of the effluent,^39^ but HCO_3_^−^ was used as the pH value. The amount of buffering agent would also affect the pH of the effluent. The optimal pH value of the anammox reaction was in the range of 6.7–8.3,^40^ and the removal of excess nitrogen load would cause the pH value in the reactor to increase seriously, thereby inhibiting the anammox reaction. It can be seen from Fig. 8 that when the influent HCO_3_^−^ concentration was less than 900 mg L^−1^, the effluent pH value was greater than 8.3, which was not suitable for the growth of anammox bacteria. When the influent HCO_3_^−^ concentration was greater than 900 mg L^−1^, the pH of the effluent could be stabilized in the optimal range of pH required for the anammox reaction. The difference in pH between the influent and the effluent was minimal, which was more conducive to the progress of the anammox reaction.

### 3.6. HCO_3_^−^ inhibition pathway on the anammox bundle fiber filler system

**Fig. 10:**
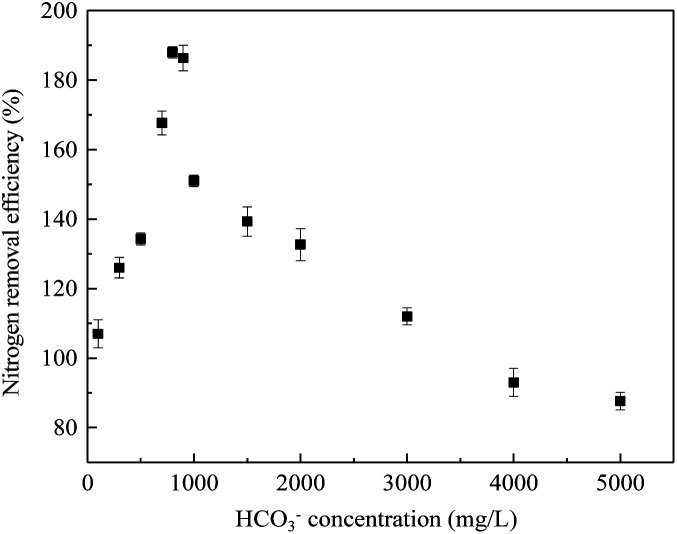
Effect of HCO_3_^−^ concentration on nitrogen removal rate in the batch test.

As shown in Fig. 10, as the HCO_3_^−^ concentration increased from 100 mg L^−1^ to 800 mg L^−1^, the removal rate of TN increased from 105 mg N (L h)^−1^ to 188 mgN (L h)^−1^. It could be explained that when the HCO_3_^−^ concentration was lower than the optimal concentration, the lack of inorganic carbon resulted in a lower TN removal rate. When the influent HCO_3_^−^ concentration was too high, although the pH remained at 7.2 (±0.2), and the FA concentration was lower than 5 mg L^−1^, it still had a negative effect on the anammox process.^41^ Therefore, it can be concluded that inorganic carbon inhibition was independent of the effect of bicarbonate on pH; therefore, it was not caused by an increase in FA. Compared with the experiment conducted under continuous conditions, the optimal HCO_3_^−^ concentration detected in the batch experiment was slightly shifted, which may be due to the increase in TN load. Rather than the constant load rate used in batch tests. Under the over-flow conditions of the filler reactor, partial FA suppression also contributed to the overall suppression, and bicarbonate was most likely to be the main contributor.

## 4. Conclusions

The anammox activity of two anammox fiber fillers is favorable. When the influent TN concentration reached 1320 mg L^−1^, two biofilm processes were not inhibited, and they still had high activity. Even with a concentration above 1650 mg L^−1^, the two kinds of filler reactors had a good impact resistance after substrate reduction, and the bundle filler showed better performance. Moreover, the curtain fiber and bundle fillers had different ways of interception of anammox bacteria. The curtain packing was highly hydrophilic, but the packing was susceptible to the impact of ingress of water. However, the bundle filler was strong with toughness, and the fibers were not easy to fall off, which was more conducive to the proliferation and aggregation of anammox bacteria. High-throughput sequencing revealed *Candidatus* Kuenenia was the dominant genus in the two biofilms, and a higher content was observed in the bundle filler (35.9%) than in the curtain filler (25.9%), which showed better efficiency and tolerance to high NO_2_^−^–N concentrations. When the influent HCO_3_^−^ concentration was 900 mg L^−1^, the bundled fiber filler had the highest removal efficiency. The inhibition can be quickly released by reducing the HCO_3_^−^ concentration. In addition, the HCO_3_^−^ inhibition mechanism was independent of pH, which resulted in high FA content.

## Conflicts of interest

The authors of this manuscript have no conflicts to declare.

## Supplementary Material
